# Modifying the conductive properties of poly(3,4-ethylenedioxythiophene) thin films in green solvents

**DOI:** 10.3389/fchem.2022.1005266

**Published:** 2022-09-12

**Authors:** Bin Hou, Chuao Ma, Sidi Li, Hongliang Liu

**Affiliations:** ^1^ Shandong Laboratory of Yantai Advanced Materials and Green Manufacturing, Yantai, China; ^2^ School of Chemistry and Chemical Engineering, Yantai University, Yantai, China

**Keywords:** electronic devices, conductive materials, PEDOT, green solvents, modification methods

## Abstract

With the rapid development of flexible electronic devices, flexible transparent conductive materials acted as the charge transport layer or electrical interconnect in the devices are of great need. As one of the representative conductive materials, poly(3,4-ethylenedioxythiophene) (PEDOT) has received more and more attention due to its high transparency in the visible region, good flexibility, especially the tunable conductivity. In order to achieve high conductivities, various of effective approaches have been adopted to modify the PEDOT thin films. However, some strategies need to be carried out in hazardous solvents, which may pollute the environment and even hinder the application of PEDOT thin films in emerging bioelectronics. Therefore, in this mini review, we focus on the discussion about the modification methods for PEDOT thin films in green solvents. According to the source of PEDOT, the modification methods of PEDOT thin films are mainly described from two aspects: 1) modification of *in-situ* PEDOT, 2) modification of PEDOT complex with poly(styrenesulfonic acid) (PEDOT:PSS). Finally, we conclude with the remaining challenges for future development on the PEDOT thin films prepared by green methods.

## Introduction

Flexible transparent conductive films have gained great attractions due to their promising application in electromagnetic shielding (Bora et al., 2019), antistatic layers (Al-Dahoudi et al., 2001), lighting displays (Yu et al., 2011), touch sensors (Worfolk et al., 2015) and bioelectronics (Berggren and Richter-Dahlfors, 2007). In recent years, various types of flexible transparent conductive films have been developed, including metallic oxides (Minami, 2008), metallic nanomaterials (Schneider et al., 2016; Hu et al., 2019), carbon nanomaterials (Kim et al., 2009; Hecht et al., 2011) and conducting polymers (Das and Prusty, 2012). Among them, conducting polymers show the merits of organic polymers with good mechanical properties, which meets the demand for flexible electronic devices. Furthermore, conducting polymers are of good biocompatibility and their properties can be fine-tuned by modulating the chemical structures and doping, which is essential to be applied in the field of biological systems (Feron et al., 2018). These unique and irreplaceable properties demonstrated that conducting polymers have a good prospect of practical application.

Poly(3,4-ethylenedioxythiophene) (PEDOT) has been one of the most widely-studied conducting polymers since 1988 when the PEDOT was first invented by Bayer AG (Jonas et al., 1988). Up to now some strategies have been developed to synthesize PEDOT, such as oxidative chemical polymerization, electrochemical polymerization and transition-metal-catalyzed polymerization (Jiang et al., 2020). As the first approach to be applied in synthesizing PEDOT, oxidative chemical polymerization is still the dominant method to prepare PEDOT now. Based on the oxidative chemical polymerization, two main types of PEDOT can be obtained. One is *in-situ* PEDOT, which is directly synthesized on the site of application and need not reprocess the PEDOT thin films. The other is PEDOT dispersion, among which PEDOT dispersed with poly(styrenesulfonic acid) (PEDOT:PSS) is the most representative. PEDOT:PSS is usually prepared and dispersed in aqueous solution first, and further processing into films is necessary for application. The properties of the PEDOT thin films are strongly dependent on the polymerization conditions and secondary treatment. Therefore, many efforts have been made to improve the electrical properties of the PEDOT thin films. However, some strategies need to be conducted in hazardous solvents such as *N*-methyl-2-pyrrolidone (NMP) (Gueye et al., 2016) and pyridine (Winther-Jensen et al., 2005), which will be harmful to the environment. Even worse, the toxic solvent residual in the PEDOT thin films may cause cytotoxicity when applied in bioelectronics. Consequently, it is essential to avoid the use of toxic solvents and modify the conductive properties of PEDOT thin films in green and sustainable solvents. In this mini review, we focus on the *in-situ* PEDOT and PEDOT:PSS, and summarize the modification methods for PEDOT thin films in green solvents. Finally, the perspectives and remaining challenges for development of high-quality PEDOT by green methods are proposed.

## Modification of PEDOT in green solvents

PEDOT is prepared by polymerization of 3,4-ethylenedioxythiophene (EDOT) monomers. To achieve the synthesis of PEDOT, EDOT monomers are first oxidated from neutral state to cationic radicals by oxidants, and then followed by polymerization. Through changing the solvents used for polymerization, reaction kinetics and PEDOT chain length can be tuned effectively. Consequently, solvents have a great effect on the conductivity of the resulting PEDOT thin film (Ha et al., 2004). In addition, secondary treatments in green solvents are also the general methods to modify the conductive properties of PEDOT thin films (Shi et al., 2015).

### Modification of *in-Situ* PEDOT

**FIGURE 1:**
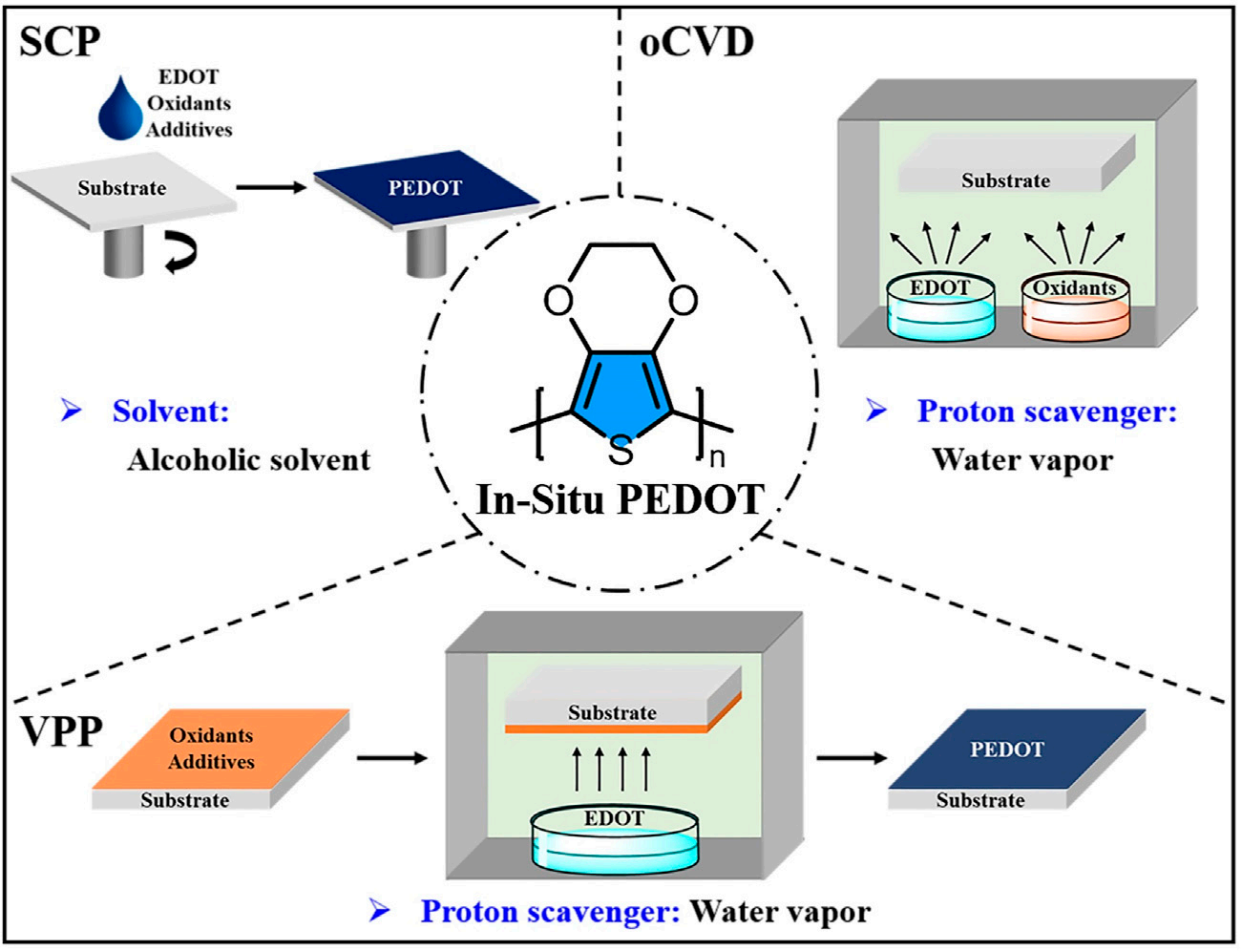
Utilization of green solvents to modify the *in-situ* PEDOT thin films prepared by SCP, VPP and oCVD.

*In-situ* PEDOT is that the EDOT monomers are polymerized *in situ* to form PEDOT under the action of oxidant. Up to now, three synthetic strategies (Figure 1) have been developed to prepare *in-situ* PEDOT: solution-cast polymerization (SCP), vapor phase polymerization (VPP) and oxidative chemical vapor deposition (oCVD). Due to the *in-situ* polymerization, the synthetic processes influence the properties of *in-situ* PEDOT greatly. Therefore, modification of *in-situ* PEDOT thin films with green solvents mainly focuses on the synthetic processes.

SCP is the original and simplest method to be applied in synthesizing PEDOT. In 1988, Bayer AG developed two reagents known as Baytron M (EDOT monomer) and Baytron C (an oxidative solution of iron (III) *p*-toluenesulfonate (Fe(OTs)_3_) in butanol) (Jonas et al., 1988). Simply spinning a mixture of these two reagents (pot-life: 10–20 min) on the substrate can obtain the PEDOT thin film. Alcoholic solvents are the common solvents used to synthesize PEDOT for SCP. Alcoholic solvents with high boiling point usually show high viscosity and hinder the reaction kinetics, which is bad for longer chain formation. However, high boiling point solvents are not easy to volatilize, thus affording a longer reaction time to form longer polymer chains, which allow higher conductivities to be achieved. Shashidhar et al. have systematically investigated the influence of the alcoholic solvents (methanol, propanol, *n*-butanol, 2-methoxyethanol, pentanol, hexanol) on the conductivities of SCP PEDOT thin films (Ha et al., 2004). Interestingly, the PEDOT thin films prepared by both high and low boiling point alcoholic solvents exhibit nearly consistent conductivities, which demonstrates that the two competing factors discussed above are balanced. Therefore, it is a little difficult to choose alcoholic solvents with high or low boiling point for the SCP method to prepare PEDOT thin films.

VPP is a general method to prepare conducting polymers, but it was not until 2003 that Kim et al. first used this strategy to synthesize PEDOT (Kim et al., 2003). A typical VPP method could be divided into three steps (Bhattacharyya et al., 2012; Brooke et al., 2017). Firstly, a solution of oxidant (e.g., FeCl_3_ (Cho et al., 2014), Fe(OTs)_3_ (Fabretto et al., 2012) and iron (III) trifluoromethanesulfonate (Fe(OTf)_3_) (Brooke et al., 2018)) with or without additive is deposited on the substrate by a casting or coating process. Then the substrate with oxidant layer is exposed to the EDOT vapor for polymerization. Bottom up (the oxidant mixture diffuses from bottom to up) (Brooke et al., 2014) and top down (the monomer diffuses from top to down) (Nair et al., 2009) are two possible mechanisms for the film growth, which is still controversial up to now. Finally, the deposited film is washed to remove impurities for purification. Water vapor in the reaction environment proved to be an effective proton scavenger and the polymerization will not occur without water vapor (Fabretto et al., 2008). However, Fe(III) oxidants have a propensity for water absorption, which easily lead to crystal formation. Therefore, high humidity during polymerization usually creates holes in the PEDOT thin film, thus decreasing the conductivity of the corresponding PEDOT thin film (Zuber et al., 2008). To overcome this problem, Fabretto et al. use an amphiphilic copolymer polyethylene glycol−polypropylene glycol−polyethylene glycol (PEG−PPG−PEG) to reserve the water and suppress crystal growth of oxidant. In addition to water storage and inhibition of crystal formation, the copolymer can reduce the effective reactivity of the oxidant, which has a similar effect to pyridine (Mueller et al., 2012). But the oxidant layer shows liquid-like state while using PEG−PPG−PEG in the polymerization process, which is different from the gel-like state for pyridine inhibitor (Evans et al., 2012). Because the PEG units have an affinity for “water” (hydrophilic domain) and the PPG moieties show an affinity for “oil” (hydrophobic domain), further studies demonstrate that the PEG/PPG ratio and molecular weight of PEG−PPG−PEG have a great effect on the conductivities of the PEDOT thin films, wherein the PEG−PPG−PEG of 5,800 Da (PEG/PPG ratio = 0.58:1) could afford a sheet-like film with the conductivity of ca. 3400 S cm^−1^ (Fabretto et al., 2012).

oCVD is another vapor deposition method to fabricate *in-situ* PEDOT, which was first developed by Gleason et al. in 2006 (Lock et al., 2006). The synthesis process of oCVD involves only one step in which the vapors of EDOT monomer and volatile oxidant (e.g., FeCl_3_ (Gharahcheshmeh and Gleason, 2019), CuCl_2_ (Im et al., 2008), SbCl_5_ (Nejati et al., 2014), VOCl_3_ (Nejati and Lau, 2011) and halogen gases (Chelawat et al., 2010)) meet and immediately undergo oxidative polymerization to obtain the PEDOT thin films on the substrate. Water vapor can also influence the properties of oCVD PEDOT, which is similar with the VPP method. While coevaporating water vapor with EDOT monomer and FeCl_3_ oxidant during the oCVD process, the water vapor will assist in dissolving FeCl_3_ oxidant. Therefore, Fe^+^ and Cl^−^ ions can be utilized efficiently, thus resulting in a relatively high doping level for PEDOT thin films when compared with the oCVD PEDOT thin films prepared with no water vapor. In addition, water vapor facilitates the stacking of the PEDOT chains perpendicular to the substrate, which is beneficial to the charge transport, thus obtaining a high conductivity of 1042 S cm^−1^ for water-assisted oCVD PEDOT thin film (Goktas et al., 2015).

### Modification of PEDOT:PSS

**FIGURE 2:**
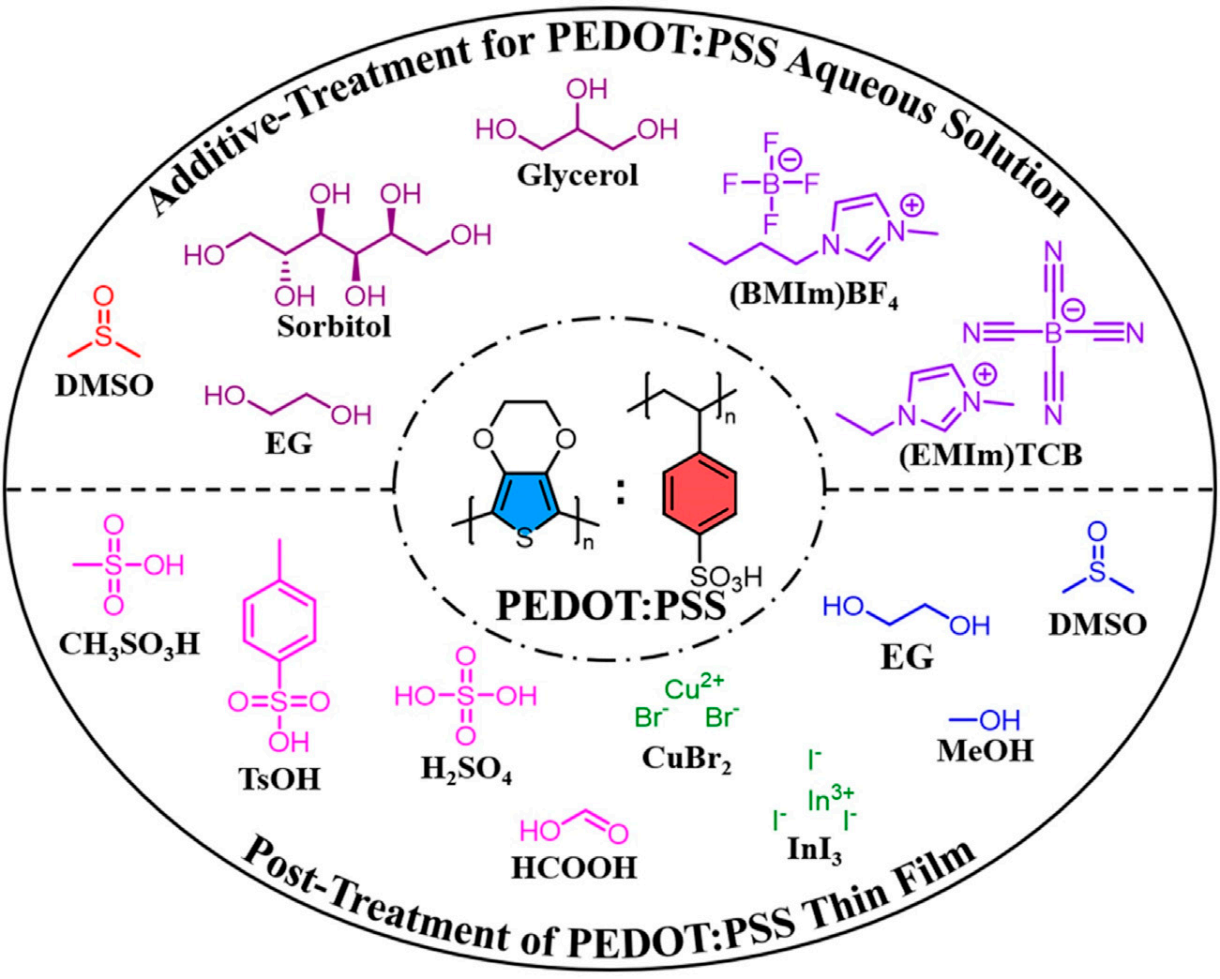
Modification of PEDOT:PSS thin films based on additive-treatment for PEDOT:PSS aqueous solution and post-treatment of PEDOT:PSS thin film in green solvents.

PEDOT:PSS is usually synthesized in an aqueous solution with peroxodisulfates (e.g., K_2_S_2_O_8_ and Na_2_S_2_O_8_) and Fe(III) salts (e.g., FeCl_3_ and Fe_2_(SO_4_)_3_) as the oxidizing reagents. Compared with aforementioned *in-situ* PEDOT, PEDOT:PSS is commercially available as a stable aqueous dispersion, in which hydrophilic and insulating PSS acts as both a counter-ion and a soluble template for hydrophobic and conducting PEDOT (Lang et al., 2009). Therefore, PEDOT:PSS is a promising conductive material, which can be easy to process by green eco-friendly techniques. Although extremely convenient, this has limited the conductivity optimization of PEDOT:PSS on the synthesis level. Therefore, additive-treatment for PEDOT:PSS aqueous solution and post-treatment of PEDOT:PSS thin film in green solvents have been two common modification methods to improve the conductivities of PEDOT:PSS thin films (Figure 2).

Green solvents such as polar solvents, polyols and ionic liquids have been used as additives for PEDOT:PSS aqueous solution to enhance the conductivities of PEDOT:PSS thin films. In 2002, Kim et al. first studied the effects of polar solvents on conductivity of the PEDOT:PSS thin film (Kim et al., 2002). While adding dimethyl sulfoxide (DMSO) in PEDOT:PSS aqueous solution, the conductivity increased from 0.8 S cm^−1^ to 80 S cm^−1^, which is more remarkable than addition of *N*,*N*-dimethylformamide (DMF) and tetrahydrofuran (THF). Since then, DMSO has been widely used as an additive to improve the conductivity of PEDOT:PSS (Lee et al., 2014; Lim et al., 2014). Polyols like ethylene glycol (EG) (Wichiansee and Sirivat, 2009), glycerol (Lee et al., 2010) and sorbitol (Onorato et al., 2010) are also added into PEDOT:PSS aqueous solution to enhance the conductivity, wherein EG is a widely-used additive. It was proved that addition of EG can increase the carrier mobility and carrier density of PEDOT:PSS thin films, thus increasing the conductivity (Wei et al., 2013). Ionic liquids are another additive for PEDOT:PSS aqueous solution to improve the conductivity of PEDOT:PSS thin films. Due to no volatility, ionic liquids can remain in the PEDOT:PSS thin films, which is different from the high boiling point solvents. In 2007, a series of ionic liquids was first envisaged as additives to enhance the conductivities of PEDOT:PSS thin films, wherein 1-butyl-3-methylimidazolium tetrafluoroborate ((BMIm)BF_4_) afforded the highest conductivity of 136 S cm^−1^ (Döbbelin et al., 2007). When compared with (BMIm)BF_4_ (287 S cm^−1^), 1-ethyl-3-methylimidazolium tetracyanoborate ((EMIm)TCB) is a better additive which makes the PEDOT:PSS thin film exhibit a conductivity of 2084 S cm^−1^ (Badre et al., 2012).

Apart from additive-treatment for PEDOT:PSS aqueous solution, post-treatment of PEDOT:PSS thin film in green solvents is also a common modification method to improve the conductivities. Polar solvents such as methanol (Alemu et al., 2012), EG (Okuzaki et al., 2009) and DMSO (Ouyang et al., 2004) have been widely used to post-treat PEDOT:PSS thin film. The hydrophilicity and dielectric constant of the alcohols have a great effect on the conductivity enhancement. While using methanol for post-treatment of PEDOT:PSS thin film, the conductivity increases from 0.3 to 1362 S cm^−1^, which is better than using ethanol and propanol (Alemu et al., 2012). Aqueous solutions of salt and zwitterion are also applied in post-treatment of PEDOT:PSS thin film. Ouyang et al. has systematically studied the cation effect of salts on the conductivity of PEDOT:PSS thin film. The result shows that salts with cations of positive soft parameter (Cu^2+^, Ag^+^, and In^3+^) can significantly enhance the conductivity of the PEDOT:PSS thin film, while the ones whose cations have negative soft parameter (Li^+^, Na^+^, Mg^2+^, and Ni^2+^) have negligible effect (Xia and Ouyang, 2009). Further studies on the anions of salts demonstrate that the conductivity enhancement is relevant to the acid dissociation constants of the anions. A salt with higher dissociation can benefit the association of the anions with PEDOT^+^, thus leading to the more significant conductivity enhancement (Xia and Ouyang, 2010). Acid aqueous solution is another choice for post-treatment of PEDOT thin films. Both strong acids like H_2_SO_4_ (Xia et al., 2012) and weak acids like methanesulfonic acid (Ouyang, 2013) and formic acid (Mengistie et al., 2014) have been studied for conductivity improvement of PEDOT:PSS thin films. For example, Kim et al. have reported the solution-processed crystalline formation in PEDOT:PSS via H_2_SO_4_ post-treatment (Kim et al., 2014). The concentrated H_2_SO_4_ treatment induces a significant structural rearrangement in the PEDOT:PSS with the removal of PSS and leads to the formation of crystallized nanofibrils via a charge-separated transition mechanism. Therefore, a conductivity of 4380 S cm^−1^ is obtained for PEDOT:PSS thin film after post-treatment with concentrated H_2_SO_4_.

In fact, treatment of PEDOT:PSS is not limited to using only one method or reagent. In order to achieve high conductivities, combination of these strategies has been utilized to treat the PEDOT:PSS. For instance, Kim et al. prepared PEDOT:PSS thin films with the highest conductivity of 1418 S cm^−1^ through adding EG into the PEDOT:PSS aqueous solution and subsequent post-treatment of PEDOT:PSS thin film with an EG bath (Kim et al., 2011). Subsequently, Pipe et al. adopt the similar method to investigate the PEDOT:PSS (DMSO-mixed) thin films post-treated with an EG bath (Kim et al., 2013). In addition, post-treatment of PEDOT:PSS thin film with a mixture of solvents is also a general method to improve the conductivities of PEDOT:PSS thin films. Luo et al. post-treat PEDOT:PSS thin films with a mixed solution of DMSO and (BMIm)BF_4_ (Luo et al., 2013). The results show that all the PEDOT:PSS thin films post-treated with the mixed solution exhibit higher conductivities compared with the pristine films. Kumar et al. find that the conductivity of the PEDOT:PSS thin film post-treated with a mixed solution of *p*-toluenesulfonic acid (TsOH) and DMSO can increase to ca. 3500 S cm^−1^ (Mukherjee et al., 2014).

## Conclusion and perspective

As one of the most widely-studied conducting polymers, PEDOT shows the merits of good flexibility, high transparency in the visible region and tunable conductivity. The conductivities of the PEDOT thin films are strongly dependent on the deposition conditions and secondary treatments. Therefore, many methods have been developed to improve the conductive properties of the PEDOT thin films. In this mini review, we summarize the modification of two types of PEDOT (*in-situ* PEDOT and PEDOT:PSS) in green solvents. The modification methods of *in-situ* PEDOT are mainly introduced from the aspect of synthetic strategies (SCP, VPP and oCVD), while the modifications of PEDOT:PSS are presented according to treatment methods (additive-treatment for PEDOT:PSS aqueous solution and post-treatment of PEDOT:PSS thin film). Although significant progress has been made in the conductivity of PEDOT, there are still some challenges. First, strategies are urgently needed that can afford a PEDOT thin film prepared by dispersion methods with a high conductivity comparable to the *in-situ* PEDOT due to the green eco-friendly process of PEDOT dispersions. In addition, microstructure plays a crucial role on the conductivity of PEDOT, but there is still a lack of effective and green methods to control the stacking orientation of PEDOT chains in the PEDOT thin film. Finally, the conductive mechanism of PEDOT is controversial, which needs further study to guide the modification of PEDOT thin films by green non-pollution approaches.

## References

[B1] Al-Dahoudi N. Bisht H. Göbbert C. Krajewski T. Aegerter M. A. (2001). Transparent conducting, anti-static and anti-static–anti-glare coatings on plastic substrates. Thin Solid Films 392, 299–304. 10.1016/S0040-6090(01)01047-1

[B2] Alemu D. Wei H.-Y. Ho K.-C. Chu C.-W. (2012). Highly conductive PEDOT:PSS electrode by simple film treatment with methanol for ITO-free polymer solar cells. Energy Environ. Sci. 5, 9662–9671. 10.1039/C2EE22595F

[B3] Badre C. Marquant L. Alsayed A. M. Hough L. A. (2012). Highly conductive poly(3, 4-ethylenedioxythiophene):poly(styrenesulfonate) films using 1-ethyl-3-methylimidazolium tetracyanoborate ionic liquid. Adv. Funct. Mat. 22, 2723–2727. 10.1002/adfm.201200225

[B4] Berggren M. Richter-Dahlfors A. (2007). Organic bioelectronics. Adv. Mat. 19, 3201–3213. 10.1002/adma.200700419

[B5] Bhattacharyya D. Howden R. M. Borrelli D. C. Gleason K. K. (2012). Vapor phase oxidative synthesis of conjugated polymers and applications. J. Polym. Sci. B. Polym. Phys. 50, 1329–1351. 10.1002/polb.23138

[B6] Bora P. J. Anil A. G. Vinoy K. J. Ramamurthy P. C. (2019). Outstanding absolute electromagnetic interference shielding effectiveness of cross-linked PEDOT:PSS film. Adv. Mat. Interfaces 6, 1901353. 10.1002/admi.201901353

[B7] Brooke R. Cottis P. Talemi P. Fabretto M. Murphy P. Evans D. (2017). Recent advances in the synthesis of conducting polymers from the vapour phase. Prog. Mat. Sci. 86, 127–146. 10.1016/j.pmatsci.2017.01.004

[B8] Brooke R. Fabretto M. Hojati-Talemi P. Murphy P. Evans D. (2014). Evidence for ‘bottom up’ growth during vapor phase polymerization of conducting polymers. Polymer 55, 3458–3460. 10.1016/j.polymer.2014.06.055

[B9] Brooke R. Franco-Gonzalez J. F. Wijeratne K. Pavlopoulou E. Galliani D. Liu X. (2018). Vapor phase synthesized poly(3, 4-ethylenedioxythiophene)-trifluoromethanesulfonate as a transparent conductor material. J. Mat. Chem. A 6, 21304–21312. 10.1039/C8TA04744H

[B10] Chelawat H. Vaddiraju S. Gleason K. (2010). Conformal, conducting poly(3, 4-ethylenedioxythiophene) thin films deposited using bromine as the oxidant in a completely dry oxidative chemical vapor deposition process. Chem. Mat. 22, 2864–2868. 10.1021/cm100092c

[B11] Cho B. Park K. S. Baek J. Oh H. S. Koo Lee Y.-E. Sung M. M. (2014). Single-crystal poly(3, 4-ethylenedioxythiophene) nanowires with ultrahigh conductivity. Nano Lett. 14, 3321–3327. 10.1021/nl500748y 24848306

[B12] Das T. K. Prusty S. (2012). Review on conducting polymers and their applications. Polym. Plast. Technol. Eng. 51, 1487–1500. 10.1080/03602559.2012.710697

[B13] Döbbelin M. Marcilla R. Salsamendi M. Pozo-Gonzalo C. Carrasco P. M. Pomposo J. A. (2007). Influence of ionic liquids on the electrical conductivity and morphology of PEDOT:PSS films. Chem. Mat. 19, 2147–2149. 10.1021/cm070398z

[B14] Evans D. Fabretto M. Mueller M. Zuber K. Short R. Murphy P. (2012). Structure-directed growth of high conductivity PEDOT from liquid-like oxidant layers during vacuum vapor phase polymerization. J. Mat. Chem. 22, 14889–14895. 10.1039/C2JM32281A

[B15] Fabretto M. V. Evans D. R. Mueller M. Zuber K. Hojati-Talemi P. Short R. D. (2012). Polymeric material with metal-Like conductivity for next generation organic electronic devices. Chem. Mat. 24, 3998–4003. 10.1021/cm302899v

[B16] Fabretto M. Zuber K. Hall C. Murphy P. (2008). High conductivity PEDOT using humidity facilitated vacuum vapour phase polymerisation. Macromol. Rapid Commun. 29, 1403–1409. 10.1002/marc.200800270

[B17] Feron K. Lim R. Sherwood C. Keynes A. Brichta A. Dastoor P. C. (2018). Organic bioelectronics: Materials and biocompatibility. Int. J. Mol. Sci. 19, 2382. 10.3390/ijms19082382 PMC6121695 30104515

[B18] Gharahcheshmeh M. H. Gleason K. K. (2019). Device fabrication based on oxidative chemical vapor deposition (oCVD) synthesis of conducting polymers and related conjugated organic materials. Adv. Mat. Interfaces 6, 1801564. 10.1002/admi.201801564

[B19] Goktas H. Wang X. Ugur A. Gleason K. K. (2015). Water-assisted vapor deposition of PEDOT thin film. Macromol. Rapid Commun. 36, 1283–1289. 10.1002/marc.201500069 25882241

[B20] Gueye M. N. Carella A. Massonnet N. Yvenou E. Brenet S. Faure-Vincent J. (2016). Structure and dopant engineering in PEDOT thin films: Practical tools for a dramatic conductivity enhancement. Chem. Mat. 28, 3462–3468. 10.1021/acs.chemmater.6b01035

[B21] Ha Y. H. Nikolov N. Pollack S. K. Mastrangelo J. Martin B. D. Shashidhar R. (2004). Towards a transparent, highly conductive poly(3, 4-ethylenedioxythiophene). Adv. Funct. Mat. 14, 615–622. 10.1002/adfm.200305059

[B22] Hecht D. S. Hu L. Irvin G. (2011). Emerging transparent electrodes based on thin films of carbon nanotubes, graphene, and metallic nanostructures. Adv. Mat. 23, 1482–1513. 10.1002/adma.201003188 21322065

[B23] Hu H. Wang S. Wang S. Liu G. Cao T. Long Y. (2019). Aligned silver nanowires enabled highly stretchable and transparent electrodes with unusual conductive property. Adv. Funct. Mat. 29, 1902922. 10.1002/adfm.201902922

[B24] Im S. G. Kusters D. Choi W. Baxamusa S. H. Van De Sanden M. C. M. Gleason K. K. (2008). Conformal coverage of poly(3, 4-ethylenedioxythiophene) films with tunable nanoporosity via oxidative chemical vapor deposition. ACS Nano 2, 1959–1967. 10.1021/nn800380e 19206437

[B25] Jiang Y. Liu T. Zhou Y. (2020). Recent advances of synthesis, properties, film fabrication methods, modifications of poly(3, 4-ethylenedioxythiophene), and applications in solution-processed photovoltaics. Adv. Funct. Mat. 30, 2006213. 10.1002/adfm.202006213

[B26] Jonas F. Heywang G. Schmidtberg W. (1988). Novel polythiophenes, process for their preparation, and their use. German Patent No 3,813,589. München: German Patent and Trade Mark Office.

[B27] Kim G. H. Shao L. Zhang K. Pipe K. P. (2013). Engineered doping of organic semiconductors for enhanced thermoelectric efficiency. Nat. Mat. 12, 719–723. 10.1038/nmat3635 23644522

[B28] Kim J. Kim E. Won Y. Lee H. Suh K. (2003). The preparation and characteristics of conductive poly(3, 4-ethylenedioxythiophene) thin film by vapor-phase polymerization. Synth. Metall. 139, 485–489. 10.1016/S0379-6779(03)00202-9

[B29] Kim J. Y. Jung J. H. Lee D. E. Joo J. (2002). Enhancement of electrical conductivity of poly(3, 4-ethylenedioxythiophene)/poly(4-styrenesulfonate) by a change of solvents. Synth. Metall. 126, 311–316. 10.1016/S0379-6779(01)00576-8

[B30] Kim K. S. Zhao Y. Jang H. Lee S. Y. Kim J. M. Kim K. S. (2009). Large-scale pattern growth of graphene films for stretchable transparent electrodes. Nature 457, 706–710. 10.1038/nature07719 19145232

[B31] Kim N. Kee S. Lee S. H. Lee B. H. Kahng Y. H. Jo Y.-R. (2014). Highly conductive PEDOT:PSS nanofibrils induced by solution-processed crystallization. Adv. Mat. 26, 2268–2272. 10.1002/adma.201304611 24338693

[B32] Kim Y. H. Sachse C. Machala M. L. May C. Müller-Meskamp L. Leo K. (2011). Highly conductive PEDOT:PSS electrode with optimized solvent and thermal post-treatment for ITO-free organic solar cells. Adv. Funct. Mat. 21, 1076–1081. 10.1002/adfm.201002290

[B33] Lang U. Müller E. Naujoks N. Dual J. (2009). Microscopical investigations of PEDOT:PSS thin films. Adv. Funct. Mat. 19, 1215–1220. 10.1002/adfm.200801258

[B34] Lee M.-W. Lee M.-Y. Choi J.-C. Park J.-S. Song C.-K. (2010). Fine patterning of glycerol-doped PEDOT:PSS on hydrophobic PVP dielectric with inkjet for source and drain electrode of OTFTs. Org. Electron. 11, 854–859. 10.1016/j.orgel.2010.01.028

[B35] Lee S. H. Park H. Son W. Choi H. H. Kim J. H. (2014). Novel solution-processable, dedoped semiconductors for application in thermoelectric devices. J. Mat. Chem. A 2, 13380–13387. 10.1039/C4TA01839G

[B36] Lim K. Jung S. Lee S. Heo J. Park J. Kang J.-W. (2014). The enhancement of electrical and optical properties of PEDOT:PSS using one-step dynamic etching for flexible application. Org. Electron. 15, 1849–1855. 10.1016/j.orgel.2014.04.014

[B37] Lock J. P. Im S. G. Gleason K. K. (2006). Oxidative chemical vapor deposition of electrically conducting poly(3, 4-ethylenedioxythiophene) films. Macromolecules 39, 5326–5329. 10.1021/ma060113o

[B38] Luo J. Billep D. Waechtler T. Otto T. Toader M. Gordan O. (2013). Enhancement of the thermoelectric properties of PEDOT:PSS thin films by post-treatment. J. Mat. Chem. A 1, 7576–7583. 10.1039/C3TA11209H

[B39] Mengistie D. A. Ibrahem M. A. Wang P.-C. Chu C.-W. (2014). Highly conductive PEDOT:PSS treated with formic acid for ITO-free polymer solar cells. ACS Appl. Mat. Interfaces 6, 2292–2299. 10.1021/am405024d 24460075

[B40] Minami T. (2008). Present status of transparent conducting oxide thin-film development for indium-tin-oxide (ITO) substitutes. Thin Solid Films 516, 5822–5828. 10.1016/j.tsf.2007.10.063

[B41] Mueller M. Fabretto M. Evans D. Hojati-Talemi P. Gruber C. Murphy P. (2012). Vacuum vapour phase polymerization of high conductivity PEDOT: Role of PEG-PPG-PEG, the origin of water, and choice of oxidant. Polymer 53, 2146–2151. 10.1016/j.polymer.2012.03.028

[B42] Mukherjee S. Singh R. Gopinathan S. Murugan S. Gawali S. Saha B. (2014). Solution-processed poly(3, 4-ethylenedioxythiophene) thin films as transparent conductors: Effect of p-toluenesulfonic acid in dimethyl sulfoxide. ACS Appl. Mat. Interfaces 6, 17792–17803. 10.1021/am504150n 25230160

[B43] Nair S. Hsiao E. Kim S. H. (2009). Melt-welding and improved electrical conductivity of nonwoven porous nanofiber mats of poly(3, 4-ethylenedioxythiophene) grown on electrospun polystyrene fiber template. Chem. Mat. 21, 115–121. 10.1021/cm8029449

[B44] Nejati S. Lau K. K. S. (2011). Chemical vapor deposition synthesis of tunable unsubstituted polythiophene. Langmuir 27, 15223–15229. 10.1021/la203318f 22047472

[B45] Nejati S. Minford T. E. Smolin Y. Y. Lau K. K. S. (2014). Enhanced charge storage of ultrathin polythiophene films within porous nanostructures. ACS Nano 8, 5413–5422. 10.1021/nn500007c 24840296

[B46] Okuzaki H. Harashina Y. Yan H. (2009). Highly conductive PEDOT/PSS microfibers fabricated by wet-spinning and dip-treatment in ethylene glycol. Eur. Polym. J. 45, 256–261. 10.1016/j.eurpolymj.2008.10.027

[B47] Onorato A. Invernale M. A. Berghorn I. D. Pavlik C. Sotzing G. A. Smith M. B. (2010). Enhanced conductivity in sorbitol-treated PEDOT–PSS. Observation of an *in situ* cyclodehydration reaction. Synth. Metall. 160, 2284–2289. 10.1016/j.synthmet.2010.08.021

[B48] Ouyang J. (2013). Solution-processed PEDOT:PSS films with conductivities as indium tin oxide through a treatment with mild and weak organic acids. ACS Appl. Mat. Interfaces 5, 13082–13088. 10.1021/am404113n 24308924

[B49] Ouyang J. Xu Q. Chu C.-W. Yang Y. Li G. Shinar J. (2004). On the mechanism of conductivity enhancement in poly(3, 4-ethylenedioxythiophene):poly(styrene sulfonate) film through solvent treatment. Polymer 45, 8443–8450. 10.1016/j.polymer.2004.10.001

[B50] Schneider J. Rohner P. Thureja D. Schmid M. Galliker P. Poulikakos D. (2016). Electrohydrodynamic nanodrip printing of high aspect ratio metal grid transparent electrodes. Adv. Funct. Mat. 26, 833–840. 10.1002/adfm.201503705

[B51] Shi H. Liu C. Jiang Q. Xu J. (2015). Effective approaches to improve the electrical conductivity of PEDOT:PSS: A review. Adv. Electron. Mat. 1, 1500017. 10.1002/aelm.201500017

[B52] Wei Q. Mukaida M. Naitoh Y. Ishida T. (2013). Morphological change and mobility enhancement in PEDOT:PSS by adding co-solvents. Adv. Mat. 25, 2831–2836. 10.1002/adma.201205158 23606373

[B53] Wichiansee W. Sirivat A. (2009). Electrorheological properties of poly(dimethylsiloxane) and poly(3, 4-ethylenedioxy thiophene)/poly(stylene sulfonic acid)/ethylene glycol blends. Mater. Sci. Eng. C 29, 78–84. 10.1016/j.msec.2008.05.018

[B54] Winther-Jensen B. Breiby D. W. West K. (2005). Base inhibited oxidative polymerization of 3, 4-ethylenedioxythiophene with iron(III) tosylate. Synth. Metall. 152, 1–4. 10.1016/j.synthmet.2005.07.085

[B55] Worfolk B. J. Andrews S. C. Park S. Reinspach J. Liu N. Toney M. F. (2015). Ultrahigh electrical conductivity in solution-sheared polymeric transparent films. Proc. Natl. Acad. Sci. U. S. A. 112, 14138–14143. 10.1073/pnas.1509958112 26515096 PMC4655535

[B56] Xia Y. Ouyang J. (2010). Anion effect on salt-induced conductivity enhancement of poly(3, 4-ethylenedioxythiophene):poly(styrenesulfonate) films. Org. Electron. 11, 1129–1135. 10.1016/j.orgel.2010.04.007

[B57] Xia Y. Ouyang J. (2009). Salt-induced charge screening and significant conductivity enhancement of conducting poly(3, 4-ethylenedioxythiophene):poly(styrenesulfonate). Macromolecules 42, 4141–4147. 10.1021/ma900327d

[B58] Xia Y. Sun K. Ouyang J. (2012). Solution-processed metallic conducting polymer films as transparent electrode of optoelectronic devices. Adv. Mat. 24, 2436–2440. 10.1002/adma.201104795 22488584

[B59] Yu Z. Niu X. Liu Z. Pei Q. (2011). Intrinsically stretchable polymer light-emitting devices using carbon nanotube-polymer composite electrodes. Adv. Mat. 23, 3989–3994. 10.1002/adma.201101986 21796688

[B60] Zuber K. Fabretto M. Hall C. Murphy P. (2008). Improved PEDOT conductivity via suppression of crystallite formation in Fe(III) tosylate during vapor phase polymerization. Macromol. Rapid Commun. 29, 1503–1508. 10.1002/marc.200800325

